# Mismatch Repair Deficiency in Esophageal Squamous Cell Carcinoma: An Underrecognized Biomarker for Immunotherapy Response

**DOI:** 10.7759/cureus.82939

**Published:** 2025-04-24

**Authors:** Carlos E Bonilla, Vaneza Ávila-Rodríguez, Paola Jiménez-Vásquez, Magda Jimena Vargas Diaz, Silvia Guerrero

**Affiliations:** 1 Gastrointestinal and Neuroendocrine Tumors Unit, Luis Carlos Sarmiento Angulo Cancer Treatment and Research Center (CTIC), Bogota, COL; 2 GIGA Research Group, Luis Carlos Sarmiento Angulo Cancer Treatment and Research Center (CTIC), Bogota, COL; 3 Internal Medicine and Hospitalization Unit, Luis Carlos Sarmiento Angulo Cancer Treatment and Research Center (CTIC), Bogota, COL; 4 Pathology Unit, Luis Carlos Sarmiento Angulo Cancer Treatment and Research Center (CTIC), Bogota, COL; 5 Gastrointestinal and Neuroendocrine Tumors Unit, uis Carlos Sarmiento Angulo Cancer Treatment and Research Center (CTIC), Bogota, COL

**Keywords:** cancer immunotherapy, mmr deficient, msi-high, pulmonary metastases, squamous esophageal cancer

## Abstract

Mismatch repair deficiency (dMMR) and high microsatellite instability (MSI-H) are known predictors of response to immune checkpoint inhibitors in gastrointestinal malignancies, often seen in adenocarcinomas. Their role in esophageal squamous cell carcinoma (ESCC) is less studied, as these alterations were historically considered rare in this subtype.

We report the case of a 74-year-old man with stage IVB proximal ESCC, presenting with bilateral lung metastases and mediastinal lymphadenopathy. Immunohistochemistry revealed dMMR with loss of PMS2 expression and a PD-L1 Combined Positive Score (CPS) of 1. After palliative radiotherapy for dysphagia, he received chemoimmunotherapy with 5-fluorouracil, cisplatin, and nivolumab. Within two months, he experienced symptom improvement, and imaging after four cycles demonstrated a partial response with marked reduction in pulmonary metastases.

This case highlights the value of testing for dMMR and MSI-H in ESCC, a subtype where these alterations have traditionally been considered uncommon. Growing evidence points to a higher prevalence than expected, especially in non-White populations, suggesting that routine dMMR/MSI-H testing in advanced ESCC could help identify patients who might benefit from immunotherapy and support more personalized, effective treatment strategies.

## Introduction

Esophageal cancer is a significant global health challenge, with high mortality driven by late-stage diagnosis and limited treatment options [1]. According to GLOBOCAN 2022, it ranks as the seventh leading cause of cancer-related mortality worldwide [2]. Surveillance, Epidemiology, and End Results (SEER) data in the United States report a five-year survival rate of only 21.7% across all stages, reflecting its poor prognosis [3]. Among its histological subtypes, esophageal squamous cell carcinoma (ESCC) accounts for approximately 85% of cases, predominant in high-risk regions like East Asia and Africa [4]. Patients typically present with progressive dysphagia, unintentional weight loss, and heartburn, often indicating locally advanced disease at diagnosis [1].

Immunotherapy with immune checkpoint inhibitors (ICIs) has improved outcomes in esophageal cancer, yet challenges in patient selection persist [5]. Biomarkers like high PD-L1 expression help select patients with greater benefit, but they do not identify all responders [5,6]. Other biomarkers, such as mismatch repair deficiency (dMMR) and high microsatellite instability (MSI-H), are established predictors of ICI response in solid tumors, enhancing tumor immunogenicity through increased mutational burden [7-9]. These markers are routinely tested in gastrointestinal adenocarcinomas, such as colorectal and esophagogastric cancers, but are rarely evaluated in ESCC, where they are considered uncommon [6].

The Cancer Genome Atlas (TCGA) study, analyzing 90 ESCC and 72 esophageal adenocarcinoma (EAC) samples from Western and Eastern populations but lacking Native American representation, found MSI-H in only one adenocarcinoma case (1.4%) and none in ESCC (0%) [10]. However, other studies in diverse regions suggest a potentially higher frequency, indicating an underestimation of dMMR/MSI-H’s role in ESCC [11,12].

Here, we present a patient with metastatic ESCC, low positive PD-L1 expression, and dMMR who achieved a partial response to chemoimmunotherapy. This case, alongside emerging data, highlights the need for molecular epidemiology studies in diverse populations and supports routine dMMR/MSI-H testing in advanced ESCC to guide personalized treatment.

## Case presentation

In June 2023, a 74-year-old male farmer from a cold, mountainous region of Colombia presented with a two-month history of progressive dysphagia, hoarseness, fatigue, and unintentional weight loss of 6 kg. He had no known personal or family history of cancer, nor traditional risk factors such as smoking, alcohol consumption, obesity, or gastroesophageal reflux disease. However, he reported habitual consumption of hot beverages and foods, a common practice in rural, cold-climate communities in Colombia, which may be associated with an increased risk of esophageal cancer.

**Figure 1:**
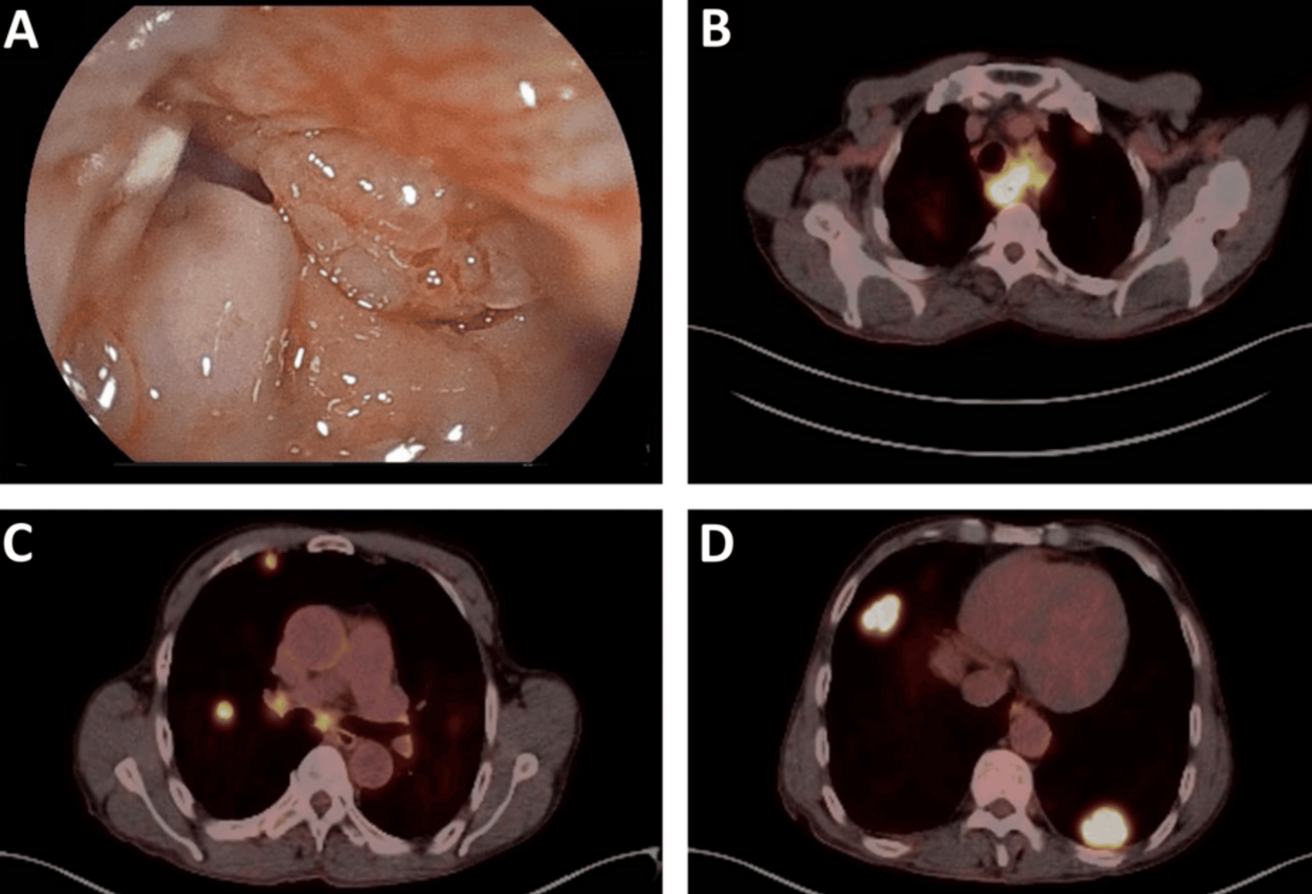
Diagnostic endoscopy and staging PET-CT imaging of dMMR proximal esophageal squamous cell carcinoma (A) Esophagogastroduodenoscopy image obtained at diagnosis in June 2023, showing a circumferential, stenosing, and rigid mass located immediately below the cricopharyngeal muscle, at 20 cm from the dental arch, consistent with esophageal carcinoma. (B) Positron emission tomography-computed tomography (PET-CT) scan (axial view) for staging, demonstrating hypermetabolic esophageal thickening at the level of T2-T4 (SUVmax 12.7), extending to the left upper paratracheal space with soft tissue density, and focal hypermetabolism in a portion of the left internal jugular vein (SUVmax 8.1). (C) PET-CT scan (coronal view) for staging, revealing bilateral mediastinal hypermetabolic lymphadenopathies (SUVmax ranging from 6.0 to 8.0) and several hypermetabolic pulmonary nodules. (D) PET-CT scan (axial view) for staging, showing dominant hypermetabolic metastases, including a 30-mm lesion in the middle lobe of the right lung (SUVmax 10.3) and a 33-mm lesion in the posterior basal segment of the left lung (SUVmax 11.9). dMMR: Mismatch repair deficiency

An upper digestive endoscopy revealed a nearly obstructive, stenotic tumor in the cervical esophagus, 18 cm from the dental arch. FDG PET-CT imaging showed a hypermetabolic primary esophageal tumor spanning T2 to T4 levels, accompanied by mediastinal lymphadenopathy and multiple bilateral lung metastases, the largest measuring 33 mm in the posterior basal segment of the left lung. Figures 1A-1D display images from upper digestive endoscopy and positron emission tomography-computed tomography (PET-CT).

**Figure 2:**
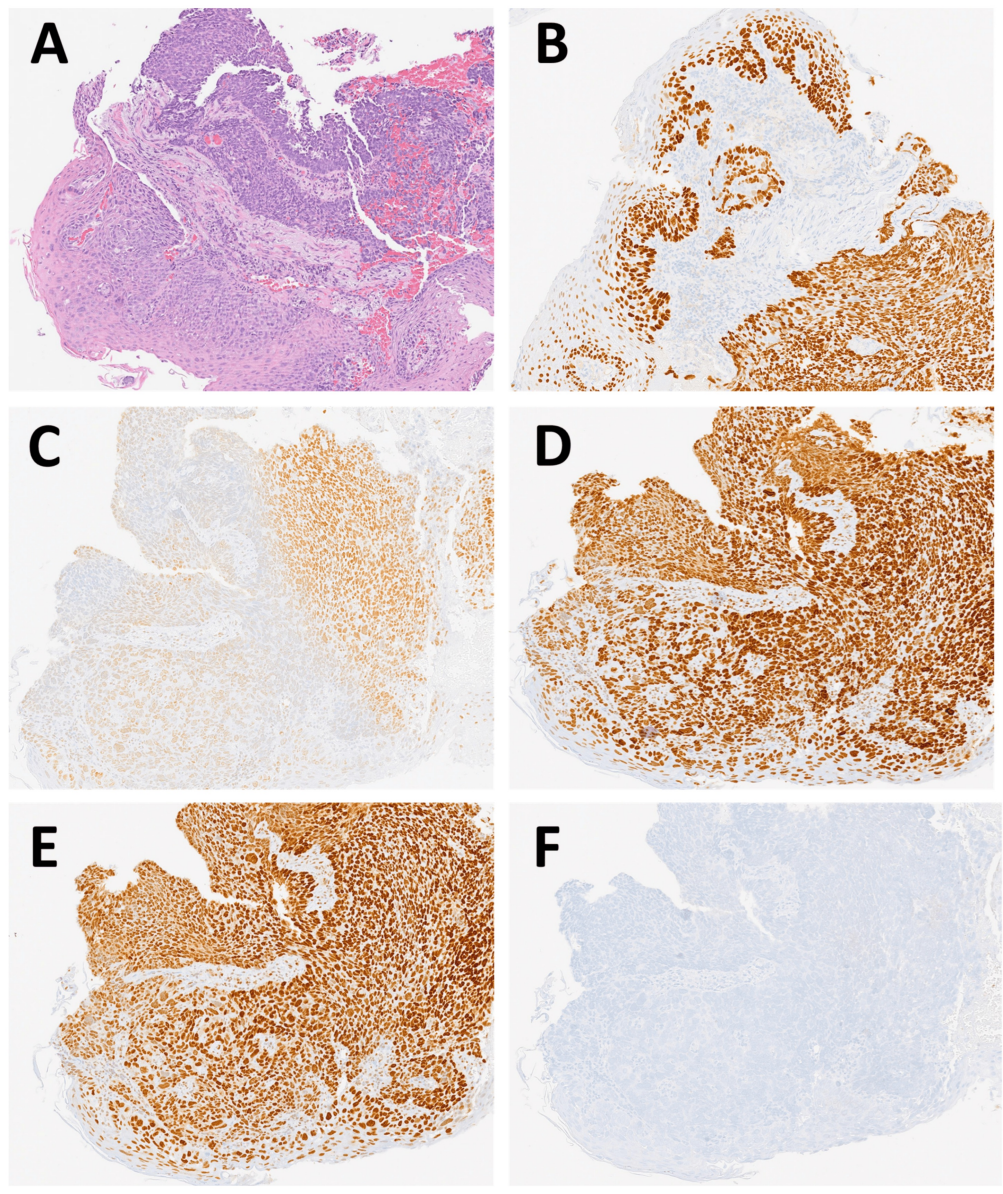
Histopathological and immunohistochemical evaluation of esophageal biopsies confirming dMMR status (magnification, 10x) (A) Hematoxylin and eosin (H&E) stain demonstrating a disorganized epithelial proliferation with marked nuclear atypia and focal necrosis. (B) Immunohistochemical staining for p63, which confirms squamous epithelial differentiation through strong nuclear positivity. (C) Patchy expression of MLH1. Retained nuclear staining for MSH2 (D) and MSH6 (E). (F) Complete loss of nuclear staining for PMS2. dMMR: Mismatch repair deficiency

An esophageal biopsy performed in October 2023 confirmed ESCC, staged as IVB, cT4aN3M1G3. Immunohistochemistry showed positivity for CKAE1/AE3, p40, and p63, with a Ki67 proliferation index of 100%, PD-L1 Combined Positive Score (CPS) of 1, and dMMR due to isolated loss of PMS2 expression. Figures 2A-2F show the immunohistochemistry on the biopsy, with loss of PMS2 expression.

**Figure 3:**
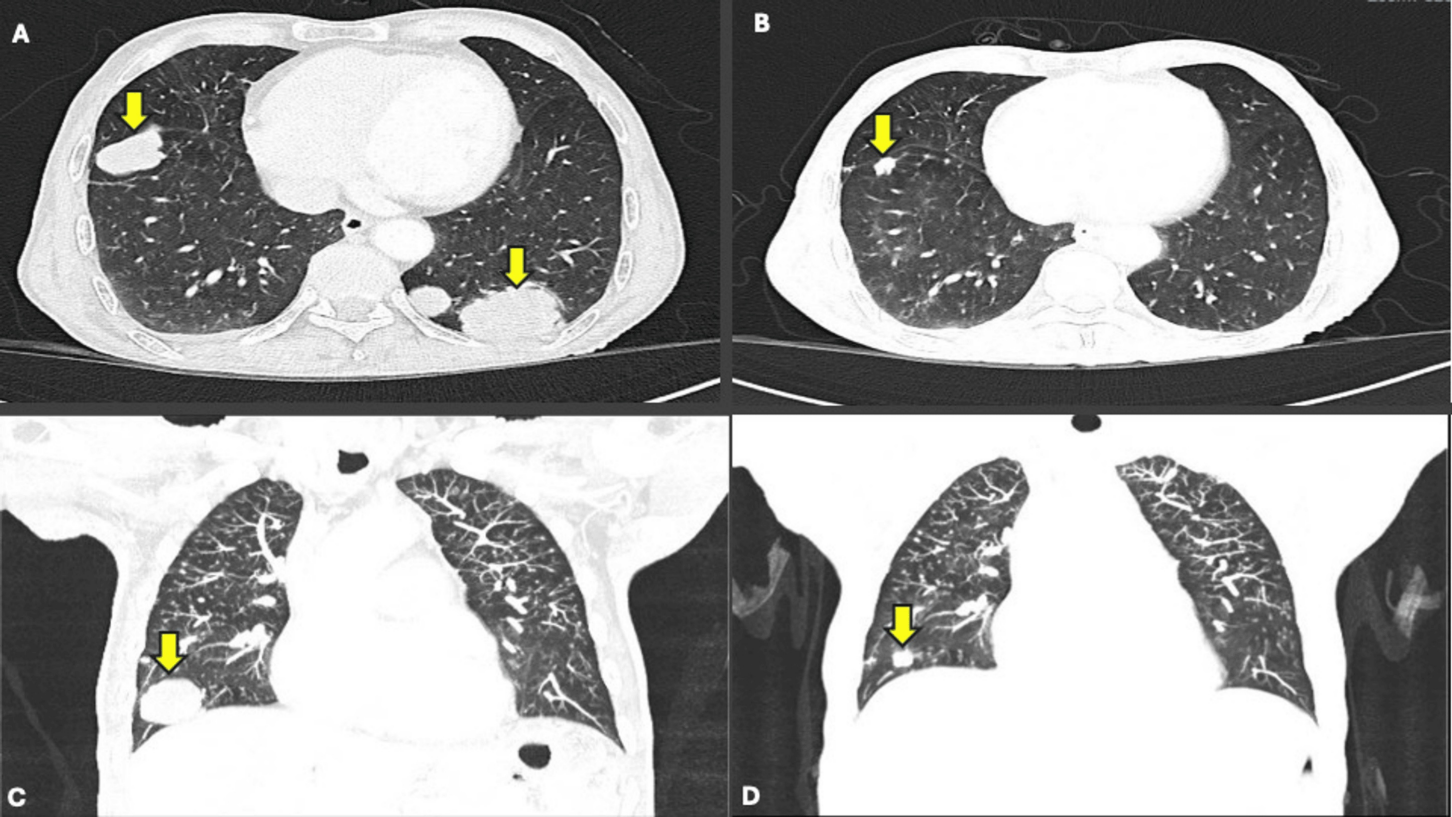
Thoracic CT scans before and after four cycles of chemoimmunotherapy (A) Baseline axial CT scan from January 2024 showing the two largest pulmonary metastases (yellow arrows) in the posterior segment of the right lower lobe and the posterior basal segment of the left lower lobe. (B) Follow-up axial CT scan from July 2024 demonstrating a marked reduction in the size of both metastases (yellow arrows), indicating a partial radiologic response. No new metastases or infiltrative changes are observed. (C) Baseline coronal CT scan from January 2024 confirming the two largest pulmonary metastases (yellow arrows) in the right and left lower lobes. (D) Follow-up coronal CT scan from July 2024 showing a significant reduction in the size of both metastases (yellow arrows), with no new metastases or infiltrative changes.

To manage dysphagia, he received palliative radiotherapy (30 Gy in 10 fractions) in November 2023. Due to persistent dysphagia, he underwent percutaneous gastrostomy placement in January 2024. Given his dMMR status, first-line systemic therapy with 5-fluorouracil, cisplatin, and nivolumab was initiated in February 2024. His symptoms improved rapidly, and by July 2024, after four cycles, a thoracic CT revealed a partial response, with a notable reduction in both the size and number of lung metastases. Figures 3A-3D show thoracic CT scans before and after four cycles of chemoimmunotherapy.

**Figure 4:**
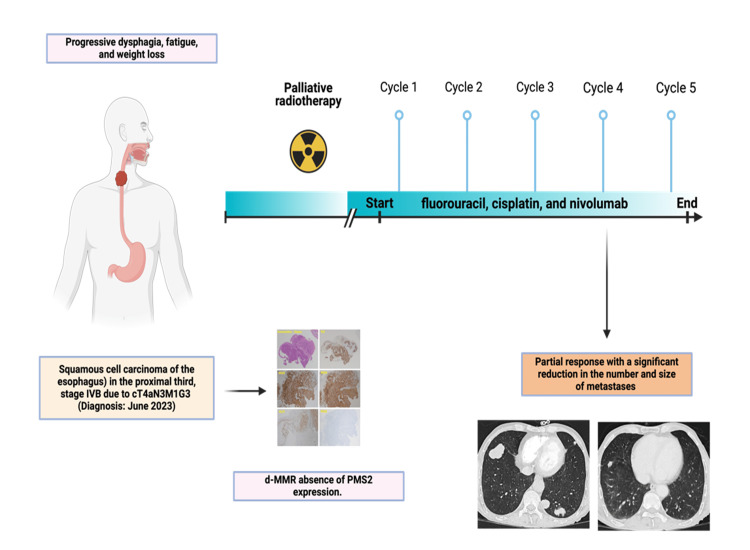
Timeline of diagnosis and treatment for a patient with stage IVB dMMR esophageal squamous cell carcinoma Image credits: BioRender (https://www.biorender.com). dMMR: Mismatch repair deficiency

The patient has since continued chemoimmunotherapy with sustained improvement, maintaining a fully functional status. Figure 4 shows the timeline of diagnosis and treatment for this patient with stage IVB dMMR ESCC. 

## Discussion

Esophageal cancer represents a significant global health burden, with approximately 511,000 new cases and 445,000 deaths annually, ranking as the seventh leading cause of cancer-related mortality worldwide [2]. Data from the U.S. SEER program indicate a five-year cancer-specific survival rate of 21.7% across all stages, dropping to 3.7% for metastatic cases, which comprise 38% of diagnoses [3]. Among all esophageal cancers, ESCC constitutes about 85% of the cases, especially in high-incidence regions such as East Asia and parts of Africa [4]. 

Standard treatment for advanced ESCC typically involves chemotherapy regimens such as 5-fluorouracil plus cisplatin, yielding a median overall survival (OS) of nine to 12 months [1]. Although recent advances with PD-1 inhibitors have improved outcomes, survival gains remain modest, underscoring the need for better predictive biomarkers and personalized treatment strategies.

MSI-H and dMMR are emerging biomarkers in ESCC, though their prevalence and clinical significance are underexplored compared to other gastrointestinal cancers such as colorectal and gastric adenocarcinoma [7,8]. Biologically, dMMR disrupts the repair of mismatched base pairs during DNA replication, resulting in MSI-H, characterized by aberrant shifts in the length of microsatellite regions-highly conserved genomic sequences prone to frameshift mutations. This instability increases tumor mutational burden (TMB) and neoantigen production, thereby enhancing tumor immunogenicity and responsiveness to ICIs [9].

Studies have reported varying frequencies of dMMR and MSI-H in ESCC. The Cancer Genome Atlas (TCGA) study on esophageal cancer, which included 90 ESCC and 72 EAC samples from Western and Eastern populations but lacked Native American representation, identified MSI-H in only one EAC case (1.4% of EAC samples) and none of the ESCC samples (0%)[10]. In contrast, studies in Asian populations have reported higher frequencies in ESCC. Matsumoto et al. analyzed 62 ESCC samples, identifying MSI-H in 8.1% [11]. More recently, Jiang et al. studied 484 ESCC cases, finding dMMR in 65 patients (13.4%), of whom six had isolated PMS2 loss (1.24% of all patients and 18.2% of dMMR patients) [12].

Immunotherapy with PD-1 inhibitors has transformed the treatment landscape for ESCC, particularly for patients with high PD-L1 expression [1,5]. The KEYNOTE-590 trial demonstrated that pembrolizumab plus chemotherapy in ESCC patients with a PD-L1 CPS ≥10 improved median OS to 13.9 months compared to 8.8 months with chemotherapy alone (HR 0.62, P<0.001) [13]. Similarly, the CHECKMATE-648 trial showed that nivolumab plus chemotherapy in patients with PD-L1 tumor proportion score (TPS) ≥1% (equivalent to a low CPS threshold) achieved a median OS of 13.2 months versus 10.7 months with chemotherapy alone (HR 0.74, P=0.002) [14].

However, PD-L1 expression as a biomarker has limitations, as patients with low PD-L1 CPS, such as the one in this case, may still respond to immunotherapy if dMMR/MSI-H is present. The KEYNOTE-158 trial confirmed that MSI-H strongly predicts immunotherapy response across multiple cancer types, with an objective response rate of 34.3%, but it excluded ESCC, limiting its direct applicability to this disease [15].

Although ESCC-specific data on immunotherapy response in dMMR/MSI-H patients are limited, the predictive value of these markers is supported by their role in increasing TMB and immunogenicity, as well as by studies confirming their predictive benefit across multiple cancers with this alteration, thereby benefiting this subgroup [8,9,15].

The case presented here demonstrates a remarkable response to chemoimmunotherapy in a patient with metastatic ESCC, dMMR due to isolated PMS2 loss, and a low but positive PD-L1 CPS of 1. Despite the low PD-L1 CPS, the patient’s dMMR status likely contributed to a high TMB, driving the favorable response to nivolumab plus chemotherapy, consistent with Jiang et al.’s findings of improved survival in PMS2-deficient ESCC [12]. This outcome challenges the reliance on PD-L1 as the sole biomarker for immunotherapy in ESCC and underscores the need for routine dMMR/MSI-H testing. Implementing such testing could identify a subset of ESCC patients-potentially 8%-13% based on prevalence data - who may benefit from immunotherapy, even with low PD-L1 expression, thus improving survival outcomes [11,12]. However, challenges such as the cost of molecular testing and access to immunotherapy in resource-constrained settings must be addressed to ensure equitable application. These findings advocate for broader molecular profiling in ESCC to guide personalized treatment and optimize the use of PD-1 inhibitors in clinical practice.

## Conclusions

This case of metastatic dMMR proximal ESCC highlights the critical need to address the underutilization of mismatch repair (MMR) and MSI testing in ESCC, where these alterations are often dismissed as rare. The scarcity of molecular epidemiological data, particularly in underrepresented populations, likely underestimates the prevalence of dMMR/MSI-H. Notably, our patient achieved a significant partial response to chemoimmunotherapy, with substantial tumor reduction despite low PD-L1 expression, consistent with the established predictive value of dMMR/MSI-H for benefit from ICIs in solid tumors. We suggest routine MMR/MSI testing in patients with advanced ESCC and urge further research to define the molecular landscape of ESCC, particularly in diverse populations.
